# Preoperative nutritional risk and adverse perioperative outcomes in children with congenital choledochal cysts: a retrospective cohort study

**DOI:** 10.3389/fped.2026.1773325

**Published:** 2026-03-20

**Authors:** Xiaoyang Liu, Yixuan Wang, Jiamin Li, Yujie Zhang, Jichang Han, Dong Sun, Qiongqian Xu, Xue Ren, Dongming Wang, Jian Wang, Aiwu Li

**Affiliations:** 1 Department of Pediatric Surgery, Qilu Hospital of Shandong University, Jinan, Shandong, China; 2 Department of Pediatric Surgery, The First Affiliated Hospital of Shandong First Medical University & Shandong Provincial Qianfoshan Hospital, Jinan, Shandong, China

**Keywords:** children, congenital choledochal cyst, nutritional risk, perioperative outcomes, STRONGkids

## Abstract

**Background:**

Congenital choledochal cysts (CCC) are rare biliary anomalies associated with significant morbidity. The impact of preoperative nutritional status on surgical outcomes in pediatric CCC patients remains unclear. This study aimed to investigate this relationship, using a validated nutritional risk screening tool to stratify patients.

**Methods:**

We conducted a retrospective cohort study of pediatric patients who underwent CCC excision with Roux-en-Y hepaticojejunostomy at a single center between January 2011 and September 2025. Nutritional risk was assessed within 24 h of admission using the Screening Tool for Risk on Nutritional Status and Growth (STRONG_kids_). Patients were categorized into moderate malnutrition risk group (MR) and high malnutrition risk group (HR). Propensity score matching (PSM) was employed to balance baseline characteristics. Perioperative outcomes were compared between groups.

**Results:**

Among 208 included patients, 107 were stratified as HR and 101 as MR before PSM. After PSM, 91 matched pairs were analyzed. The HR group had significantly lower weight-for-age (WAZ), height-for-age (HAZ), and BMI-for-age z-scores (BAZ), along with lower preoperative hemoglobin, albumin, total protein, and higher bilirubin and GGT levels. Postoperatively, the HR group experienced longer abdominal drainage duration, delayed gastrointestinal recovery, higher inflammatory markers (WBC, CRP), worse liver function markers, and lower albumin and lymphocyte counts. The overall complication rate was significantly higher in the HR group, primarily driven by a greater incidence of cholangitis.

**Conclusion:**

Preoperative high nutritional risk, identified by the STRONG_kids_ screening tool, is strongly associated with adverse perioperative outcomes in children undergoing CCC surgery. Routine nutritional screening may facilitate risk stratification and guide preoperative optimization.

## 1 Introduction

Congenital choledochal cysts (CCC), or congenital biliary dilatation, are a developmental malformation of the biliary system characterized by congenital dilation of the common bile duct, with possible accompanying dilation of the intrahepatic bile ducts (1). CCC demonstrate marked geographic heterogeneity, with Japan accounting for 67% of Asian cases. Incidence exceeds 1/1,000 live births in Asian populations, contrasting with 1/100,000–150,000 in Western cohorts (2). Despite being primarily benign lesions, CCC retain critical clinical significance due to potentially fatal complications including biliary obstruction, recurrent cholangitis, gallstone formation, and pancreatitis (3). The cornerstone of CCC management involves complete surgical cyst excision with Roux-en-Y hepaticojejunostomy (4, 5).

Nutrition is fundamental for maintaining physiological homeostasis and promoting development. Catabolic states precipitate rapid depletion of bodily reserves, resulting in compromised immune function and elevated rates of morbidity and mortality (6). Research has indicated that 18%–60% of pediatric surgical patients present with malnutrition at admission, while 20%–50% experience further deterioration in nutritional status during hospitalization (7). Nutritional deficiency in pediatric surgical patients exerts clinically notable adverse effects on outcomes, manifesting as prolonged hospitalization duration, elevated risks for mortality, hospital readmission rates, and postoperative complications (8, 9).

While the identification of malnutrition and its associated risks is well-established in adult surgical patients, evidence on its prevalence and prognostic impact in pediatric surgical patients remains scarce. Given their inherently higher metabolic demands, young children are particularly predisposed to increased catabolism and nutritional disturbances during physiological stress. Pediatric patients with CCC frequently develop malnutrition secondary to hepatic dysfunction, manifesting as reduced nutrient intake, impaired fat and fat-soluble vitamin absorption, and disrupted nutrient metabolism (10). Early identification and intervention for nutritional risks in children with CCC are thus clinically imperative.

The Screening Tool for Risk on Nutritional Status and Growth (STRONG_kids_) (11), developed by Hulst et al., is a nutritional risk screening instrument designed for pediatric populations. However, its applicability in children with CCC remains underexplored. This study aimed to investigate the relationship between preoperative nutritional status and perioperative outcomes in pediatric patients with CCC, with the goal of providing evidence to underscore the importance of nutritional assessment, which may inform future holistic treatment strategies for this population.

## 2 Patients and methods

This study was conducted in accordance with the ethical standards of the Institutional Review Board of Qilu Hospital, Shandong University (Approval No. KYLL-2025SL-419-02). The presentation of this work follows the STROBE (Strengthening the Reporting of Observational studies in Epidemiology) criteria (12).

### 2.1 Patients

This retrospective study was performed at the Department of Pediatric Surgery, Qilu Hospital. A total of 208 patients with CCC underwent operations at our institution from January 2011 to September 2025. All surgical procedures were performed by an experienced senior pediatric surgeon.

The inclusion criteria were as follows:

1. Pediatric patients diagnosed with CCC at our institution's Department of Pediatric Surgery between January 2011 and September 2025 were included based on preoperative imaging, including computed tomography (CT),and magnetic resonance cholangiopancreatography (MRCP).
2. All surgical candidates underwent choledochal cyst excision with Roux-en-Y hepaticojejunostomy.
3. Complete clinical data were available for all included patients.

Exclusion criteria were as follows:

1. Secondary biliary dilation due to biliary stones, strictures, or tumors, as confirmed by medical history and imaging.
2. Non-definitive surgical management such as cholecystostomy or operative cholangiography.
3. Incomplete clinical data, including missing medical records or diagnostic results.

### 2.2 Methods

#### 2.2.1 Nutritional risk screening

In this study, nutritional risk screening for CCC patients was performed using the STRONG_kids_ screening tool, administered by experienced pediatric surgeons within 24 h of admission. This assessment encompasses four domains: subjective clinical evaluation, reduced nutritional intake, weight loss or impaired growth, and disease severity. The scoring system classifies patients as follows: 0 points indicating low nutritional risk, 1–3 points indicating moderate risk, and 4–5 points indicating high risk (Table 1).

**Table 1:** STRONG_kids_: screening tool for risk on nutritional status and growth.

Screening risk of malnutrition	Score	
Assess following items within 24 h after admission and once a week thereafter		
1. Subjective clinical assessment (1 point).	No	Yes → 1
Is the patient in a poor nutritional status judged by subjective clinical assessment (diminished subcutaneous fat and/or muscle mass and/or hollow face)?		
2. High risk disease (2 points).	No	Yes → 2
Is there an underlying illness with a risk of malnutrition or expected major surgery		
3. Nutritional intake and losses (1 point).	No	Yes → 1
Are one of the following items present?		
Excessive diarrhoea (≥5 per day) and/or vomiting (>3 times/day) the last few days? Reduced food intake during the last few days before admission (not including fasting for an elective procedure or surgery)? Pre-existing dietetically advised nutritional intervention? Inability to consume adequate nutritional intake because of pain		
4. Weight loss or poor weight gain? (1 point)	No	Yes → 1
Is there weight loss or no weight gain (infants < 1year) during the last few week/months?		

For the purpose of this screening, CCC was considered an “underlying illness with a risk of malnutrition”. Thus, all patients received a score of 2 for Item 2: High-risk disease. Based on the nutritional risk screening assessment, the pediatric subjects were stratified into two groups: those with moderate malnutrition risk and those with high malnutrition risk.

#### 2.2.2 Data collection

Baseline data encompassed age, gender, American Society of Anesthesiologists (ASA) classification, and comorbidities.

Clinical information was collected from the hospital electronic medical records, with missing data was addressed using multiple imputation.

Patient-related factors included gender, age at surgery, weight, and height. Surgical and clinical details consisted of hospital length of stay (LOS), postoperative hospital LOS, hospitalization costs, surgical methods, operative time, intraoperative blood loss, shape of the cyst, protein plugs of the distal common bile duct, common hepatic duct stenosis, blood transfusion, reoperation, duration of abdominal drainage, and time to gastrointestinal recovery. The criteria for abdominal drain removal were met when the daily output was serous in nature and less than 30 mL for two consecutive days, in the absence of signs of bile leakage or infection. Gastrointestinal recovery was defined as the return of bowel sounds, passage of flatus, and the ability to tolerate oral liquid or semi-liquid diet without nausea, vomiting, or abdominal distension.

Comorbidities were defined as conditions unrelated to CCC, such as Epstein–Barr virus (EBV) infection or hepatic hemangioma. Postoperative complications were defined as any deviations from the normal postoperative recovery course, occurring from the time of surgery through hospitalization and subsequent follow-up, including but not limited to calculi, cholangitis, pancreatitis, intestinal adhesion or obstruction, and anastomotic stenosis. Complications were identified from medical records and were reviewed and adjudicated by two independent researchers, with any discrepancies resolved by a senior surgeon.

Pre- and postoperative laboratory parameters included: white blood cell count (WBC), C-reactive protein (CRP), hemoglobin (Hb), platelet count (PLT), lymphocyte count (LYM#), total bilirubin (TBIL), direct bilirubin (DBIL), indirect bilirubin (IBIL), alanine aminotransferase (ALT), aspartate aminotransferase (AST), gamma-glutamyl transferase (GGT), total protein (TP), albumin (ALB), creatinine (Cr), blood urea nitrogen (BUN), serum potassium (K), serum sodium (Na), serum chlorine (Cl), serum calcium (Ca), serum phosphorus (P) and serum magnesium (Mg).

#### 2.2.3 Statistical analyses

The determination of weight-for-age z-scores (WAZ), height-for-age z-scores (HAZ), and BMI-for-age z-scores (BAZ) in pediatric patients was performed using the WHO Anthro software.

Data processing and statistical analysis were performed using SPSS version 27.0. The normality of continuous variables was assessed using the Shapiro–Wilk test, and homogeneity of variances was evaluated with Levene's test. Normally distributed measurement data were expressed as mean ± standard deviation and compared between two groups using the *t*-test when variances were equal. For data with skewed distribution or unequal variances, the Mann–Whitney *U* test was used for comparisons. Skewed data were presented as median (interquartile range). Categorical data were summarized as frequencies and percentages and compared using the chi-square test. A *p*-value of less than 0.05 was considered statistically significant.

Given the retrospective cohort design of this study, baseline characteristics may be unevenly distributed between the MR and HR groups, potentially introducing confounding factors. Propensity score matching (PSM) was performed using a logistic regression model with selected covariates. A caliper width of 0.05 standard deviations of the logit propensity score was used.

To identify potential risk factors for postoperative complications, univariate logistic regression analyses were performed for all clinically relevant preoperative variables. Given the limited number of complication events (*n* = 22), to avoid model overfitting, no more than three variables were entered into the final multivariate model in accordance with the events-per-variable principle. Odds ratios (ORs) with 95% confidence intervals (CIs) were calculated. Model calibration was assessed using the Hosmer–Lemeshow goodness-of-fit test. A two-sided *p* value < 0.05 was considered statistically significant.

## 3 Result

### 3.1 Baseline characteristics

**Figure 1:**
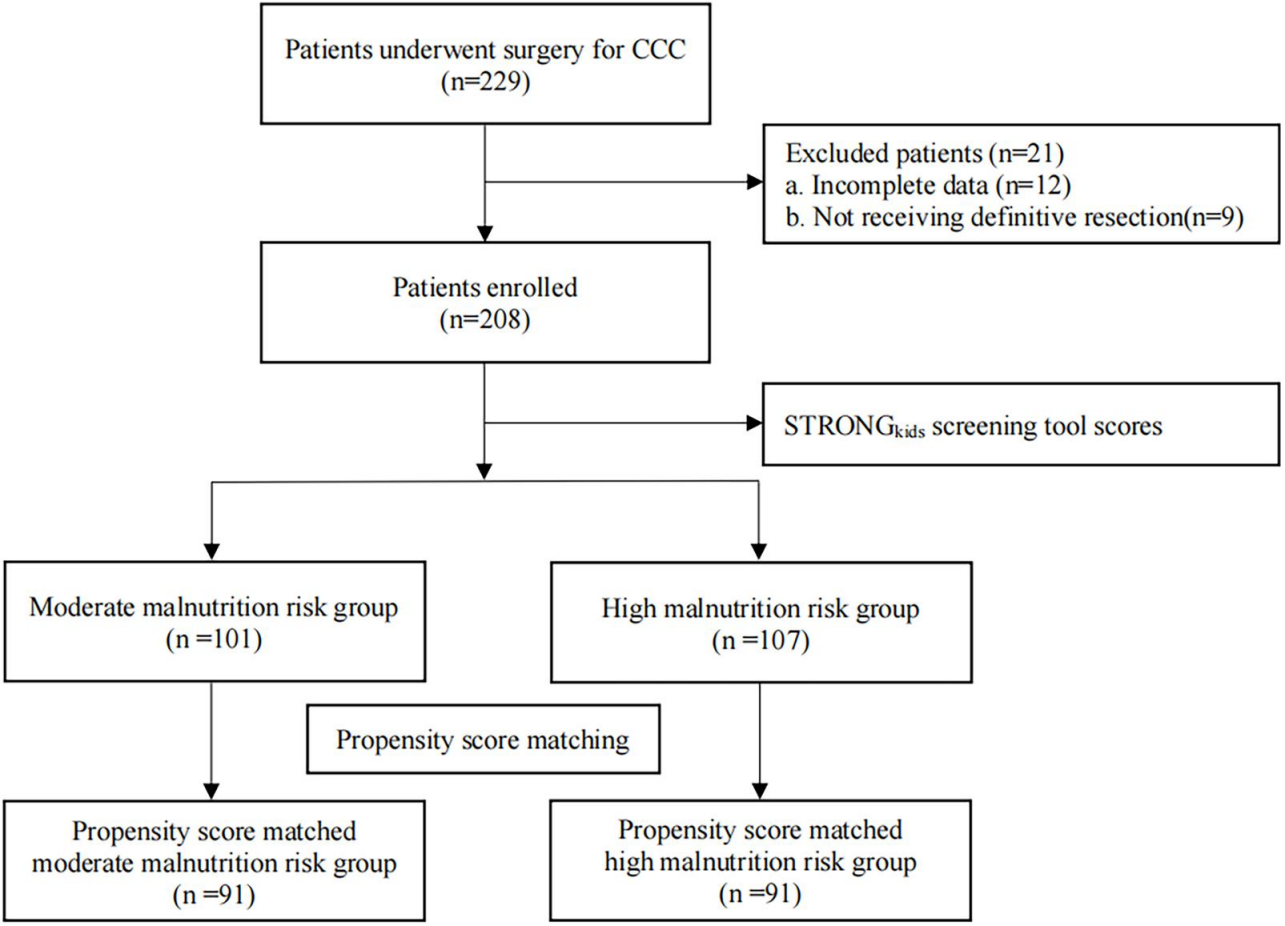
Study flow chart.

The study flow chart was summarized in Figure 1. During the study period, 229 pediatric patients with CCC underwent surgery at our center, among whom 12 were excluded due to incomplete data and 9 were excluded for not receiving definitive resection.

A total of 208 children were included in this study, with a mean age of 42.09 ± 41.70 months. The cohort comprised 148 females (71.15%) and 60 males (28.85%). Nutritional risk screening was conducted within 24 h of admission using the STRONG_kids_ screening tool. Scores were distributed as follows: 0 patients scored 0; 0 scored 1; 49 scored 2; 52 scored 3; 96 scored 4; and 11 scored 5. Based on these results, patients were categorized into a moderate malnutrition risk group (MR) and a high malnutrition risk group (HR). As shown in Table 2, the HR group exhibited significantly younger age (*p* = 0.006) and a higher prevalence of comorbidities (*p* = 0.037) compared to the MR group. To adjust for potential confounders, PSM was performed using a logistic regression model that incorporated age, gender, ASA classification, and comorbidities.

**Table 2:** Baseline characteristics of pediatric patients with CCC before and after PSM.

Variables	Before PSM			After PSM		
	MR	HR	*p*-value	MR	HR	*p*-value
	*n* = 101	*n* = 107		*n* = 91	*n* = 91	
Age (months)	34.00 (18.00, 60.00)	23.5 (8.25, 48.25)	0.006	32.00 (16.00, 59.00)	25.00 (8.00, 53.00)	0.194
Gender (%)			0.060			0.611
Male	23 (22.8)	37 (34.6)		22 (24.2)	25 (27.5)	
Female	78 (77.2)	70 (65.4)		69 (75.8)	66 (72.5)	
ASA (%)			0.210			0.486
I/II	87 (86.1)	98 (91.6)		79 (86.8)	82 (90.1)	
III/IV	14 (13.9)	9 (8.4)		12 (13.2)	9 (9.9)	
Comorbidities (%)			**0** **.** **037**			1
Yes	100 (99.0)	100 (93.5)		90 (98.9)	90 (98.9)	
None	1 (1.0)	7 (6.5)		1 (1.1)	1 (1.1)	
WAZ	1.09 (0.55, 1.31)	−0.73(−1.21, −0.30)	**<0** **.** **001**	1.09 (0.57, 1.43)	−0.71(−1.23, −0.30)	**<0** **.** **001**
HAZ	0.23(−0.16, 1.16)	−0.29(−0.6, 0.23)	**<0** **.** **001**	0.23(−0.16, 1.01)	−0.28(−0.52, 0.30)	**<0** **.** **001**
BAZ	0.94 (0.41, 1.77)	−0.88(−1.79, −0.27)	**<0** **.** **001**	0.90 (0.43, 1.80)	−0.94(−1.83, −0.29)	**<0** **.** **001**
WBC, ×10^9^/L	7.66 (5.88, 9.26)	9.10 (6.4, 11.89)	**0** **.** **003**	7.78 (6.57, 9.38)	8.46 (6.27, 11.39)	0.197
Hb, g/L	119.42 ± 12.63	113.54 ± 12.72	**<0** **.** **001**	118.55 ± 12.76	113.62 ± 12.92	**0** **.** **01**
PLT, ×10^9^/L	344.13 ± 109.08	387.36 ± 121.31	**0** **.** **008**	353.23 ± 110.34	384.17 ± 114.44	0.065
LYM#, ×10^9^/L	3.59 (2.50, 5.02)	4.16 (2.91, 6.34)	**0** **.** **016**	3.86 (2.58, 5.12)	3.95 (2.72, 5.89)	0.492
TBIL, μmol/L	7.60 (4.75, 20.20)	15.30 (6.70, 71.20)	**<0** **.** **001**	7.90 (4.90, 21.50)	19.10 (6.70, 71.20)	**<0** **.** **001**
DBIL, μmol/L	3.40 (1.95, 11.15)	7.10 (2.60, 39.20)	0.012	3.50 (2.00, 11.60)	8.00 (2.90, 39.20)	**0** **.** **015**
IBIL, μmol/L	3.90 (2.65, 7.46)	6.30 (3.30, 18.40)	<0.001	3.90 (2.70, 7.60)	7.10 (3.40, 19.20)	**<0** **.** **001**
ALT, U/L	43.00 (18.50, 103.00)	35.00 (15.00, 122.00)	0.53	48.00 (19.00, 104.00)	33.00 (16.00, 122.00)	0.421
AST, U/L	38.00 (25.50, 73.50)	42.00 (28.00, 93.00)	0.183	39.00 (26.00, 76.00)	42.00 (27.00, 85.00)	0.36
GGT, U/L	238.00 (94.50, 270.00)	334.65 (194.00, 334.65)	**0** **.** **001**	278.29 (94.00, 283.00)	368.34 (199.00, 368.34)	**<0** **.** **001**
ALB, g/L	43.70 (41.30, 46.00)	41.90 (38.60, 44.30)	**<0** **.** **001**	43.70 (41.30, 46.20)	41.90 (38.60, 44.70)	**0** **.** **002**
TP, g/L	63.99 ± 5.11	60.85 ± 6.90	**<0** **.** **001**	63.78 ± 4.99	60.78 ± 6.86	**<0** **.** **001**
Cr, μmol/L	29.00 (23.00, 35.50)	26.00 (22.00, 31.00)	**0** **.** **044**	29.00 (22.00, 34.00)	25.00 (22.00, 32.00)	0.191
BUN, mmol/L	3.31 (2.49, 4.22)	2.90 (2.18, 3.60)	0.144	3.30 (2.35, 4.22)	2.90 (2.15, 3.60)	0.125
K, mmol/L	4.51 (4.19, 4.83)	4.73 (4.25, 4.99)	0.053	4.52 (4.18, 4.85)	4.64 (4.18, 4.91)	0.189
Na, mmol/L	140.00 (138.00, 142.00)	139.00 (137.00, 140.00)	**0** **.** **003**	140.00 (138.00, 142.00)	139.00 (137.00, 140.00)	**0** **.** **011**
Cl, mmol/L	105.00 (103.00, 107.00)	105.00 (102.00, 107.00)	0.241	105.00 (103.00, 107.00)	105.00 (103.00, 107.00)	0.572
Ca, mmol/L	2.46 ± 0.11	2.46 ± 0.14	0.968	2.47 ± 0.11	2.45 ± 0.14	0.413
P, mmol/L	1.68 ± 0.24	1.68 ± 0.26	0.962	1.67 ± 0.24	1.69 ± 0.27	0.652
Mg, mmol/L	0.90(0.86, 0.95)	0.92(0.87, 0.98)	0.118	0.90(0.87, 0.95)	0.92(0.88, 0.98)	0.126

CCC, congenital choledochal cysts; PSM, propensity score matching; MR, moderate malnutrition risk group; HR, high malnutrition risk group; ASA, American society of anesthesiologists classification; WAZ, weight-for-age z-scores; HAZ, height-for-age z-scores; BAZ, BMI-for-age z-scores; WBC, white blood cell count; Hb, hemoglobin; PLT, platelet count; LYM#, lymphocyte count; TBIL, total bilirubin; DBIL, direct bilirubin; IBIL, indirect bilirubin; ALT, alanine aminotransferase; AST, aspartate aminotransferase; GGT, gamma-glutamyl transferase; ALB, albumin; TP, total protein; Cr, creatinine; BUN, blood urea nitrogen; K, serum potassium; Na, serum sodium; Cl, serum chlorine; Ca, serum calcium; P, serum phosphorus; Mg, serum magnesium.

The bold values indicate statistical significance (*p* < 0.05).

After PSM, 91 patients were included in each cohort. No significant intergroup differences were observed in age, sex, ASA classification, or comorbidities. The MR group demonstrated significantly higher WAZ, HAZ, and BAZ compared to the HR group (*p* < 0.001). Among preoperative laboratory parameters, hemoglobin levels were significantly elevated in the MR group (*p* = 0.01). Conversely, the MR group exhibited markedly lower levels of TBIL (*p* < 0.001), DBIL (*p* = 0.015), IBIL (*p* < 0.001), and GGT (*p* < 0.001). Furthermore, ALB (*p* = 0.002) and TP (*p* < 0.001) levels were significantly higher in the MR group than in the HR group.

### 3.2 Operative data

As shown in Table 3, the MR and HR groups showed no significant differences in surgical methods (*p* = 0.700), operative time (*p* = 0.237), intraoperative blood loss (*p* = 0.199), cyst shape (*p* = 0.835), common hepatic duct stenosis (*p* = 0.282), or packed red blood cell transfusion (*p* = 0.017).

**Table 3:** Comparison of perioperative variables between the MR and HR groups.

Variables	MR	HR	*p*-value
	*n* = 91	*n* = 91	
Surgical approach (%)			0.7
Laparoscopic	88 (96.7)	87 (95.6)	
Laparotomy	3 (3.3)	4 (4.4)	
Operative time (minutes)	225 (195, 250)	230 (205, 265)	0.237
Intraoperative blood loss (mL)	8 (5, 15)	8 (5, 10)	0.199
Shape of the cyst (%)			0.835
Cystic	77 (84.6)	78 (85.7)	
Non-cystic	14 (15.4)	13 (14.3)	
Protein plug of distal common bile duct (%)			**0** **.** **002**
Yes	6 (6.6)	21 (23.1)	
None	85 (93.4)	70 (76.9)	
Common hepatic stenosis (%)			0.282
Yes	15 (16.5)	10 (11.0)	
None	76 (83.5)	81 (89.0)	
Blood transfusion			
Fresh frozen plasma (mL)	200 (175, 400)	300 (200, 500)	**0** **.** **017**
Suspended red blood cell (U)	1.0 (0.0, 1.0)	0.8 (0.0, 1.0)	0.649
Total hospital LOS (days)	20 (16, 24)	22 (17.0, 27)	**0** **.** **026**
Postoperative hospital LOS (days)	9 (8, 10)	10 (9, 11)	**0** **.** **02**
Hospitalization costs ($)	6,394.78 (5,773.46, 7,280.58)	6,805.69 (6,464.94, 7,866.88)	**0** **.** **018**

MR, moderate malnutrition risk group; HR, high malnutrition risk group; LOS, length of stay.

The bold values indicate statistical significance (*p* < 0.05).

Compared to the MR group, however, the HR group had a higher incidence of protein plugs in the distal common bile duct (*p* = 0.002) and received more fresh frozen plasma transfusion (*p* = 0.017). Additionally, the MR group had shorter total hospital LOS (*p* = 0.026), shorter postoperative LOS (*p* = 0.02), and lower hospitalization costs (*p* = 0.018).

### 3.3 Postoperative recovery

As shown in Table 4, patients in the MR group exhibited significantly shorter abdominal drainage duration (*p* = 0.018) and faster gastrointestinal recovery (*p* = 0.004) than those in the HR group.

**Table 4:** Postoperative recovery outcomes between the MR and HR groups.

Variables	MR	HR	*p*-value
	*n* = 91	*n* = 91	
Duration of abdominal drainage (days)	8.00 (7.00, 10.00)	9.00 (8.00, 11.00)	**0** **.** **018**
Time to gastrointestinal recovery (days)	4.00 (3.00, 5.00)	4.00 (4.00, 6.00)	**0** **.** **004**
WBC, ×10^9^/L	8.75 (6.78, 10.26)	9.01 (7.89, 9.77)	**0** **.** **047**
CRP, mg/L	14.34 (8.74, 20.45)	25.55 (7.72, 26.11)	**0** **.** **05**
Hb, g/L	115.29 ± 11.384	111.53 ± 12.235	**0** **.** **033**
PLT, ×10^9^/L	320.87 ± 85.34	346.51 ± 102.18	0.068
LYM#, ×10^9^/L	2.74 (2.04, 3.18)	3.28 (2.10, 4.06)	**0** **.** **001**
TBIL, μmol/L	10.80 (6.60, 12.30)	15.60 (6.40, 17.80)	**0** **.** **012**
DBIL, μmol/L	4.60 (2.10, 5.30)	6.70 (2.30, 7.20)	**0** **.** **021**
IBIL, μmol/L	5.20 (3.78, 5.32)	7.80 (3.50, 7.90)	**<0** **.** **001**
ALT, U/L	38.00 (20.00, 41.00)	43.00 (20.00, 48.00)	0.118
AST, U/L	39.00 (29.00, 46.00)	43.00 (29.00, 54.00)	0.072
GGT, U/L	32.50 (17.75, 82.75)	72.50 (26.00, 118.00)	0.067
ALB, g/L	42.00 (38.00, 44.00)	38.00 (35.80, 40.90)	**<0** **.** **001**
TP, g/L	62.69 ± 6.60	58.62 ± 6.86	**<0** **.** **001**
Cr, μmol/L	24.00 (21.00, 31.00)	25.00 (19.50, 29.00)	0.751
BUN, mmol/L	1.97 (1.41, 2.78)	2.03 (1.33, 2.83)	0.891
K, mmol/L	4.11 (3.76, 4.47)	4.21 (3.61, 4.66)	0.752
Na, mmol/L	139.00 (137.00, 140.00)	139.00 (137.00, 141.00)	0.155
Cl, mmol/L	104.00 (102.00, 105.00)	104.00 (102.00, 107.00)	0.141
Ca, mmol/L	2.42 (2.28, 2.52)	2.35 (2.25, 2.48)	0.134
P, mmol/L	1.33 ± 0.27	1.32 ± 0.30	0.71
Mg, mmol/L	0.82 (0.76, 0.89)	0.84(0.77, 0.90)	0.645

MR, moderate malnutrition risk group; HR, high malnutrition risk group; WBC, white blood cell count; CRP, C-reactive protein; Hb, hemoglobin; PLT, platelet count; LYM#, lymphocyte count; TBIL, total bilirubin; DBIL, direct bilirubin; IBIL, indirect bilirubin; ALT, alanine aminotransferase; AST, aspartate aminotransferase; GGT, gamma-glutamyl transferase; ALB, albumin; TP, total protein; Cr, creatinine; BUN, blood urea nitrogen; K, serum potassium; Na, serum sodium; Cl, serum chlorine; Ca, serum calcium; P, serum phosphorus; Mg, serum magnesium.

The bold values indicate statistical significance (*p* < 0.05).

Laboratory findings demonstrated significantly lower postoperative levels of WBC (*p* = 0.047), CRP (*p* = 0.05), and LYM# (*p* = 0.001) in the MR group.

Regarding liver function, the MR group also showed significantly reduced levels of TBIL (*p* = 0.012), DBIL (*p* = 0.021), and IBIL (*p* < 0.001). In contrast, Hb (*p* = 0.033), ALB (*p* < 0.001) and TP (*p* < 0.001) levels were significantly higher in the MR group.

### 3.4 Postoperative complications

As detailed in Table 5, the HR group exhibited a significantly higher overall complication rate than the MR group, with 18 of the 22 complications occurring in HR patients (*p* = 0.001). Cholangitis incidence was significantly elevated in the HR group, accounting for 8 of the 9 documented cases (*p* = 0.017). No significant intergroup differences were observed for pancreatitis (8 cases, *p* = 0.47), calculi (4 cases, *p* = 0.312), intestinal obstruction (1 case, *p* = 0.316), anastomotic stenosis (2 cases, *p* = 0.155), stress ulcer (1 case, *p* = 0.316), or gastrointestinal bleeding (1 case, *p* = 0.316). Reoperations were performed in 5 patients, 4 of whom were from the HR group, with no statistically significant difference between groups (*p* = 0.174).

**Table 5:** Postoperative complication rates between the MR and HR group.

Variables	MR	HR	*p*-value
	*n* = 91	*n* = 91	
Overall postoperative complications (%)			**0** **.** **001**
Yes	4 (4.4%)	18 (19.8%)	
None	87 (95.6%)	73 (80.2%)	
Cholangitis (%)			**0** **.** **017**
Yes	1 (1.1%)	8 (8.8%)	
None	90 (98.9%)	83 (91.2%)	
Pancreatitis (%)			0.47
Yes	3 (3.3%)	5 (5.5%）	
None	88 (96.7%)	86 (94.5%)	
Calculi (%)			0.312
Yes	1 (1.1%)	3 (3.3%)	
None	90 (98.9%)	88 (96.7%)	
Intestinal obstruction (%)			0.316
Yes	0 (0%)	1 (1.1%)	
None	91 (100%)	90 (98.9%)	
Anastomotic stenosis (%)			0.155
Yes	0 (0%)	2 (2.2%)	
None	91 (100%)	89 (97.8%)	
Stress ulcer (%)			0.316
Yes	0 (0%)	1 (1.1%)	
None	91 (100%)	90 (98.9%)	
Gastrointestinal bleeding (%)			0.316
Yes	0 (0%)	1 (1.1%)	
None	91 (100%)	90 (98.9%)	
Reoperation (%)			0.174
Yes	1 (1.1%)	4 (4.4%)	
None	90(98.9%)	87(95.6%)	

MR, moderate malnutrition risk group; HR, high malnutrition risk group.

The bold values indicate statistical significance (*p* < 0.05).

To further evaluate whether nutritional parameters were independently associated with postoperative complications, binary logistic regression analysis was performed (Table 6). Univariate logistic regression analysis showed that higher TBIL levels (OR = 1.029, 95% CI = 1.019–1.040, *p* < 0.001), higher GGT levels (OR = 1.001, 95% CI = 1.000–1.003, *p* = 0.021), lower WAZ (OR = 0.268, 95% CI = 0.15–0.476, *p* < 0.001), lower ALB (OR = 0.894, 95% CI = 0.83–0.964, *p* = 0.003) and lower Hb (OR = 0.959, 95% CI = 0.926–0.993, *p* = 0.02) were significantly associated with increased risk of postoperative complications. Multivariate logistic regression analysis showed that lower WAZ (OR = 0.265, 95% CI = 0.141–0.497, *p* < 0.001) and lower ALB (OR = 0.905, 95% CI = 0.838–0.977, *p* = 0.011) were independently associated with higher complication risk.

**Table 6:** Univariate and multivariate logistic regression analysis of risk factors for postoperative complications.

Variables	Univariate logistic			Multivariate logistic		
	OR	95% CI	*p*-value	OR	95% CI	*p*-value
Age	1.004	0.994–1.014	0.437			
TBIL	1.029	1.019–1.040	<0.001			
GGT	1.001	1.000–1.003	0.021			
WAZ	0.268	0.15–0.476	<0.001	0.265	0.141–0.497	**<0** **.** **001**
ALB	0.894	0.83–0.964	0.003	0.905	0.838–0.977	**0** **.** **011**
Hb	0.959	0.926–0.993	0.02	0.971	0.932–1.011	0.149

TBIL, total bilirubin; GGT, gamma-glutamyl transferase; WAZ, weight-for-age z-scores; ALB, albumin; Hb, hemoglobin.

The bold values indicate statistical significance (*p* < 0.05).

## 4 Discussion

Our study demonstrates a significant association between preoperative nutritional risk, as identified by the STRONG_kids_ screening tool, and adverse perioperative outcomes in pediatric patients undergoing surgery for CCC. Our findings indicate that patients at high nutritional risk experienced prolonged postoperative recovery, higher inflammatory responses, more pronounced liver function impairments, and a greater incidence of overall complications, particularly cholangitis, compared to those at moderate risk.

In recent years, advancements in clinical nutrition and pediatric surgery have heightened surgeons' awareness of perioperative nutritional management in children. Previous studies have confirmed that undernutrition is a risk factor for adverse postoperative outcomes, whereas optimal nutritional status is associated with improved clinical results. Guidelines from the European Society for Clinical Nutrition and Metabolism (ESPEN) (13), American Society of Parenteral and Enteral Nutrition (ASPEN) (14), The Chinese Society of Parenteral and Enteral Nutrition (CSPEN) (15) all recommend systematic nutritional risk screening and assessment in hospitalized patients. Identifying nutritional risk before surgery is essential, underscoring the need for a rapid, simple, and accurate nutritional screening tool in clinical practice (16).

Several nutritional screening tools (NSTs), including the Pediatric Nutrition Risk Score (PNRS), Subjective Global Nutritional Assessment (SGNA), Pediatric Yorkhill Malnutrition Score (PYMS), Screening Tool for the Assessment of Malnutrition in Paediatrics (STAMP), and STRONG_kids_ screening tool, have been validated in pediatric populations (17, 18). Although there is currently no internationally standardized tool for pediatric nutritional risk screening, multiple studies have demonstrated that the STRONG_kids_ screening tool exhibited high sensitivity and specificity. Its simplicity, practicality, rapid administration, and good patient compliance make it advantageous in clinical settings (19–21). Therefore, our study adopted STRONG_kids_ screening tool as the standard tool for malnutrition risk screening.

A pivotal finding of our study is the validation of the STRONG_kids_ screening tool in the pediatric patients diagnosed with CCC. The tool effectively discriminated between patient groups, with the HR cohort showing significantly lower WAZ, HAZ, and BAZ scores. This is consistent with previous validation studies by Huysentruyt et al. (22) and Barros et al. (23), confirming its utility in identifying children with anthropometric deficits.

Beyond identifying nutritional status, our study establishes a clear link between high nutritional risk and inferior surgical outcomes. The HR group had a significantly longer duration of abdominal drainage and time to gastrointestinal recovery, contributing to their extended total hospital LOS, postoperative hospital LOS and higher hospitalization costs. Our laboratory findings further support this observation. The HR group demonstrated significantly reduced postoperative levels of ALB and TP, indicating poorer nutritional and immunological status (24–26), along with significantly elevated postoperative bilirubin levels (TBIL, DBIL, IBIL), consistent with greater cholestasis (27). The higher postoperative levels of WBC and CRP in the HR group suggest a more pronounced systemic inflammatory response to surgical stress (28). Furthermore, the significantly elevated rates of overall complications and specifically cholangitis in the HR group underscore the clinical urgency of preoperative nutritional optimization. Pre-existing malnutrition may aggravate surgical trauma, potentially increasing susceptibility to biliary infections (29).

To elucidate the independent role of nutritional parameters on perioperative outcomes, multivariate logistic regression identified both WAZ and serum ALB as independent predictors of postoperative complications. These findings collectively highlight the critical importance of preoperative nutritional assessment.

This study has several limitations inherent to its retrospective design. Although propensity score matching was employed to mitigate selection bias and balance baseline characteristics, unmeasured confounding factors may persist. Furthermore, the data were sourced from a single high-volume tertiary center and lacked long-term follow-up, which may limit the generalizability of our findings to other healthcare settings and the assessment of sustained outcomes. Additionally, the STRONG_kids_ screening tool incorporates a subjective clinical assessment, which, while practical, could introduce some interobserver variability. Furthermore, as a retrospective study, it does not establish causality or inform clinical decisions regarding surgical timing. Specifically, our data cannot determine whether delaying surgery for preoperative nutritional optimization in high-risk patients would improve outcomes. Therefore, the findings should be interpreted as identifying a high-risk cohort rather than as direct evidence to support routine postponement of surgery.

Despite these limitations, our findings hold significant clinical implications. The routine implementation of systematic nutritional risk screening using validated tools like STRONG_kids_ screening tool is imperative for children presenting with CCC. Early identification of high-risk patients should trigger a comprehensive nutritional assessment and prompt initiation of multimodal nutritional interventions, whether enteral or parenteral, aimed at optimizing metabolic reserves before surgery. Future prospective, multicenter studies are warranted to confirm the causal relationship between nutritional status and outcomes and to evaluate the impact of targeted preoperative nutritional support on mitigating perioperative risks in this vulnerable population.

In conclusion, preoperative nutritional risk, as screened by the STRONG_kids_ screening tool, is strongly associated with worse perioperative outcomes in children undergoing surgery for CCC. Integrating routine nutritional screening into the preoperative workup is a simple yet effective strategy to stratify risk, which may inform more vigilant perioperative care and could guide future research into preoperative optimization strategies.

## Data Availability

The raw data supporting the conclusions of this article will be made available by the authors, without undue reservation.
